# The Hormel Institute International Cancer Research Conference—2017 meeting report

**DOI:** 10.1038/s41698-018-0046-1

**Published:** 2018-02-09

**Authors:** Tia Rai, Ann M. Bode, Zigang Dong

**Affiliations:** The Hormel Institute, University of Minnesota, 801 16th Ave NE, Austin, MN 55912 USA

## Abstract

Despite considerable advances in our understanding of the mechanisms that contribute to cancer and improved treatment outcomes for many cancers, the burden of cancer still remains a huge issue for society. Thus, cancer researchers from around the globe must pool their resources to improve cancer care and outcomes. The 2017 Hormel Institute International Cancer Research Conference, co-sponsored by the Masonic Cancer Center, University of Minnesota, provided an opportunity for a diverse group of scientists to meet and discuss recent advances in cancer research and prevention. The 2-day conference, held in Austin, Minnesota, on June 19–20, 2017, was divided into nine scientifically driven sessions that focused broadly on fundamental cancer research, molecular mechanisms of tumor development, tumor stem cells, tumor therapeutic and preventive mechanisms, and achievements in tumor prevention and therapy.

## Introduction

The burden of cancer on society remains a huge issue worldwide. In 2015, an estimated 17.5 million cancer cases and 8.7 million cancer-related deaths occurred.^1^ Despite these statistics, considerable progress has been made in understanding of the mechanisms that contribute to cancer. These advances have been accompanied by technical breakthroughs that have improved the specific targeting of certain kinds of cancer and improved patients’ outlook and prognosis. Some of these advances include the remarkable developments in gene editing technologies (i.e., CRISPR-Cas9), which allow for targeted gene manipulations at the nucleic acid level. Progress in in silico techniques has improved both high-throughput screening technologies that have enabled the screening of thousands of compound libraries and facilitated the identification of small molecule inhibitors targeting specific cancer pathway components. Improvements in drug design technology have increased the successful synthesis of effective new molecules and enhanced the ability to tweak pre-existing molecules to result in better binding and enhanced activity. Great strides in the field of immunotherapy have resulted in the creation of better therapeutic monoclonal antibodies, which are now prescribed for several types of cancers.^2^ Our understanding of the human microbiome and the associated metabolome has also improved and is beginning to provide crucial information regarding their potentially important roles in cancer development and progression. Technological innovations, such as “next-generation” sequencing, have allowed sequencing and genetic testing to be used for diagnosis and risk assessment in standard oncology clinical practice.^3,4^ In addition, major paradigm shifts have occurred in basic thinking about cancer, including a major impetus toward precision therapies, which focus on treating specific cancer patients, rather than standard toxic therapies that have been used for decades. Conferences such as the 2017 Hormel Institute International Cancer Conference, which brought together scientists from around the world, are thus even more important because they stimulate collaboration and innovation. Utilizing novel and exciting technological advances in cancer biology will certainly improve the prognosis of this deadly and complex disease. A brief overview of the conference is provided, with presentations broadly divided by subject area and details regarding talks presented at the conference are provided in Table 1.

**Table 1:** Talks presented at the 2017 Hormel Institute International Cancer Conference

	Speaker name	Affiliation	Presentation title
1	Rajesh Agarwal	University of Colorado	Silibinin inhibits tumorigenic potential of colon cancer stem cells
2	Marc Bissonnette	The University of Chicago	Vitamin D and Losartan suppress colonic tumorigenesis in conditional Apc mouse model of colon cancer and exert differential effects on Notch signals
3	Ann M. Bode	The Hormel Institute, University of Minnesota	The Paradox of the * Wilms' Tumor 1* Gene
4	Andrew T. Chan	Massachusetts General Hospital	Aspirin and Cancer: The promise of precision chemoprevention
5	John DiGiovanni	The University of Texas at Austin	Novel approaches targeting amino acid metabolism for the prevention and treatment of prostate cancer
6	Zigang Dong	The Hormel Insitute, University of Minnesota	Precision prevention and therapy of colon cancer
7	Richard L. Eckert	University of Maryland School of Medicine	A novel vascularization mechanism and therapy target in epidermal cancer stem cells
8	Sergio Gradilone	The Hormel Institute, University of Minnesota	The primary cilium as a therapeutic target in carcinogenesis
9	Stephen S. Hecht	University of Minnesota	Tobacco smoke carcinogens and lung cancer susceptibility
10	Edward Hinchcliffe	The Hormel Institute, University of Minnesota	Histone H3.3 Ser31 phosphorylation is required to prevent chromosome instability
11	Luke Hoeppner	The Hormel Institute, University of Minnesota	Targeting dopamine and cyclic AMP-regulated phosphoprotein (DARP-32) to inhibit lung cancer growth: a novel precision therapy strategy
12	Marina K. Holz	Yeshiva University	Crosstalk between mTORC1 and estrogen signaling in breast cancer
13	Mark R. Kelley	Indiana University School of Medicine	Exploiting the Ref-1-APE1 node in cancer signaling and other diseases: From bench to clinic
14	Celina G. Kleer	University of Michigan	Investigating new genetic drivers of metaplastic breast carcinomas: role of the matricellular protein CCN6
15	Laura A. Kresty	Medical College of Wisconsin	Cranberry Proanthocyanidins inhibit esophageal adenocarcinoma in association with reduced bile acid metabolites and improved microbiome profiles
16	Andrea Macaluso	Nature Partner Journals	The Future of Open Science
17	Rebecca J. Morris	The Hormel Institute, University of Minnesota	Evidence for expression of epidermal cytokeratin and mRNA in blood and bone marrow
18	Lalita Shevde-Samant	The University of Alabama	Targeting pathways that drive epithelial plasticity and chemoresistance in cancer
19	Yong Sang Song	Seoul National University	Cholesterol and chemoresistance in ovarian cancer
20	Chung S. Yang	Rutgers University	Does vitamin E prevent or promote cancer?
21	Guang-Yu Yang	Northwestern University	Chemoprevention of colitis-induced carcinogenesis via targeting soluble epoxide hydrolase
22	Douglas Yee	Masonic Cancer Center, University of Minnesota	Disruption of insulin receptor function in breast cancer
23	Ming You	Medical College of Wisconsin	Targeting honokiol to mitochondria leads toa striking inhibitory effect on lung cancer in mice

### Advances in chemoprevention: colorectal cancer (CRC)

The old adage “prevention is better than cure” holds particularly true for cancer because once established, cancers frequently change and mutate, making them difficult to treat and cure, as they develop resistance to available therapies. Several conference speakers shared their recent work on the important topic of cancer chemoprevention for CRC. CRC is a leading cause of death for men and women in the United States, therefore it is imperative to identify means of reducing CRC incidence.^5^ Dietary components have been shown to affect growth factor signals, thus acquiring a better understanding of the mechanism of action of dietary compounds is crucial for effective cancer chemoprevention.

Dr. Marc Bissonnette presented work from his group, showing that a combination of the angiotensin II blocker, Losartan, with dietary vitamin D, inhibited colon cancer in a mouse model, and that this combination had differential effects on Notch signaling. While further studies are needed, dietary vitamin D could be a promising approach for preventing colon cancer.

Dr. Rajesh Agarwal presented work from his group on the natural compound silibinin (milk-thistle extract) and its chemopreventive role in colon cancer. Using a specially devised mouse model, his group showed that mice fed with silibinin exhibited reduced tumor size, volume and latency, a decreased number of cancer stem cells and attenuated tumor metabolic activity. Thus certain natural compounds might provide an important tool in cancer chemoprevention.

Chronic inflammation of the colon (colitis) is a known risk factor for CRC, therefore targeting colitis could help prevent colon carcinogenesis. Dr. Guang-Yu Yang discussed his work on preventing colitis-induced-carcinogenesis by targeting the soluble epoxide hydrolase (sEH). sEH converts dietary anti-inflammatory compounds into inflammatory compounds. Inhibiting sEH suppresses inflammation by attenuating NF-κB and TNF-α-induced cell adhesion molecule expression. In a mouse model, soluble sEH inhibitors decreased active colitis and carcinogenesis and affected fatty acid metabolic profiles, suggesting that sEH could be an attractive target for cancer chemoprevention.

Aspirin (salicylic acid) has been known to aid cardiovascular health for some time; however, it can also lower CRC risk. Indeed, the evidence of lowered CRC risk is so strong that the US Preventive Services Task Force (USPTF) recommends daily low dose aspirin for chronic disease prophylaxis, including CRC prevention.^6^ Dr. Andrew T. Chan presented work on the anti-CRC effects of aspirin and the development of biomarkers that could help identify patients most likely to benefit from taking aspirin for CRC prevention. This is crucial because aspirin can cause gastro–intestinal bleeding, making elucidating a risk/benefit profile imperative for individual CRC patients. This precise approach could help pave the way for a larger global effort focused on aspirin-mediated chronic disease prevention.

Dr. Zigang Dong further addressed precision CRC prevention. His group found that CRC progression is associated with elevated thromboxane A2 levels (TXA2) and identified TXA2 levels as an important CRC biomarker. They have also previously shown that TXA2 plays an important role in breast cancer.^7^ Aspirin could reduce TXA2 levels, providing mechanistic insight into its protective effects against CRC. Dr. Dong’s group has also identified other genetic targets for CRC prevention such as CDK2, COX-1, TOPK and AKT, as well as chemopreventive agents like licochalcone A (i.e., licorice root extract) and esculetin (i.e., a coumarin derivative).

### Advances in chemoprevention: other cancers

Chemopreventive approaches utilizing dietary supplements have also been evaluated for other cancers. A better understanding of how these dietary supplements are metabolized and their potential effects on our microbiome and metabolome profiles could provide valuable information in assessing their cancer chemopreventive abilities and two conference speakers directly addressed these important issues.

Previous studies suggested that vitamin E could help prevent cancer; however, large-scale trials that evaluated vitamin E and cancer prevention have had very disappointing results. Dr. Chung S. Yang and his group examined data from many trials and animal studies and identified certain factors that could determine the risk/benefit of vitamin E in cancer. These factors include the nutritional status of the patient, the dose and, most importantly, the form of vitamin E used. Vitamin E consists of a mix of α, γ and δ tocopherols. The γ and δ tocopherols are probably more effective in cancer prevention due to differences in metabolism, transport, signaling and conversion to side chain products.

Dr. Laura Kresty discussed her research on the inhibition of esophageal adenocarcinomas (EACs) by cranberry proanthocynadins (C-PAC). EAC has a terrible prognosis; however, a portion of EAC cases are preventable. Using a rat model that mimicked human reflux-induced EAC, Dr. Kresty’s group showed that rats fed C-PAC had reduced EAC, a changed bile acid profile and less inflammation in the gut microbiome. Thus C-PAC could potentially provide a valuable approach for preventing gastroesophageal reflux-linked cancer.

### Advances in fundamental cancer research—cancer cell signaling, chemoresistance, cancer metabolism and biomarker development

Another major theme of the conference focused on cancer cell signaling. Specifically, discussion emphasized a deeper understanding of cancer cell signaling that could be useful in the development of new effective treatments, understanding drug resistance mechanisms, improving existing therapies and in identifying pertinent biomarkers

Breast cancer remains a significant health issue with 316,120 new cases and 40,610 deaths estimated to occur in 2017.^8^ Several conference speakers presented their work on the issue. Dr. Marina Holz shared her work on the interactions between the mTOR pathway and estrogen-receptor (ER) signaling in breast cancer. Dr. Holz’s group recently identified an interaction between the mechanistic target of the rapamycin complex 1 receptor (mTORC1) and the ER. Understanding the mTORC1/estrogen signaling nexus has been critical for the development of FDA-approved mTOR inhibitors for the treatment of advanced ER-positive breast cancer.

Dr. Celina Kleer discussed her work on the genetic drivers of metaplastic breast carcinomas, an understudied and unusual form of invasive breast cancer. Dr. Kleer’s group identified the matricellullar protein, CCN6, as a tumor suppressor and created a CCN6 knock-out mouse model. CCN6 null mice develop metaplastic tumors that are morphologically similar to human tumors and could serve as an important model to evaluate therapies against this aggressive form of breast cancer.

Dr. Douglas Yee presented work on the role of a specific insulin receptor (InsR) in breast cancer. His group showed that insulin-stimulated growth in monolayer growth assays of endocrine-resistant cell lines decreased when they disrupted InsR function using synthetic protein ligands. While InsR has an important function in normal glucose homeostasis, cancer cells express a splice variant of the receptor (InsR-A), that is not expressed on normal cells. Therefore, the development of InsR-A specific binders could selectively target cancer cells and spare normal cells.

Dr. Yong Sang Song shared his work on the role of cholesterol in the development of drug resistance in ovarian cancer. Dr. Song’s group discovered that targeting cholesterol levels in the tumor ascites environment (TME) could help overcome drug resistance. Drug resistance could also be overcome by silencing the liver X receptor (*LXR*) gene, which is up-regulated in the TME by cholesterol and is involved in cholesterol-mediated drug resistance.

Dr. Mark Kelley discussed his work on the reduction–oxidation (redox) factor 1-apurinic/apyrimidinic endonuclease (Ref-1/APE1), a critical node for cancer cell signaling.^9^ This enzyme regulates transcription factor activation and also participates in the DNA damage response. Ref-1/APE1 is activated in several cancers and can lead to increased aggressiveness, inflammation and angiogenesis in the tumor environment. Dr. Kelley and his group showed that APX330, a drug that selectively blocks Ref-1/APE1 could kill tumor cells in 3-dimensional spheroid models, and in patient derived tumor models.

Dr. Lalita Shevde-Samant shared her work on pathways that drive epithelial plasticity and drug resistance in cancer. Cancer cells can hijack developmental signaling pathways such as the hedgehog (Hh) signaling pathway for their own purposes. Examining the role of Hh signaling in breast cancer, Dr. Shevde–Samant’s group found that tumor cells could usurp the Hh pathway to regulate macrophage polarization, creating a more favorable stromal environment for themselves.

Dr. Edward Hinchcliffe discussed his work on histone H3.3 and its role in preventing chromosome instability. Aneuploidy, caused by chromosome missegregation during mitosis, is a hallmark of cancer cell progression. Dr. Hinchcliffe’s group discovered an “aneuploidy checkpoint”, which is triggered by the phosphorylation of histone H3.3 by Chk1 kinase. Prevention of H3.3 phosphorylation by mutation or Chk1 inhibition abolished the “aneuploidy checkpoint” and increased chromosome missegregation, leading to populations of chromosomally unstable cells that could contribute to early oncogenic activity.

Dr. Rebecca Morris presented work on the expression of epidermal cytokeratins in blood and bone marrow. Cytokeratins are frequently found in the blood and bone marrow of patients with epithelial cancers and might promote metastasis. To determine if cytokeratins are expressed in adult mice, Dr. Morris’s group used fluorescence-associated cell sorting to identify cells that expressed keratin on their surface and found a small but distinct sub-population of keratin-reactive cells. In a mouse model for skin carcinogenesis, some mice developed keratin-reactive skin tumors from bone marrow-derived cells. These results could shed light on our understanding of cutaneous biology, non-melanoma skin cancer, and other epithelia and their cancers.

Dr. Ann Bode discussed work on the Wilms' tumor 1 (*WT1*) gene. *WT1* deletion is associated with the development of Wilms' tumor, a rare kidney cancer, suggesting its activity as a tumor suppressor. However, high *WT1* expression is also associated with poor prognosis in some cancers; thus *WTI* appears to function as both a tumor suppressor and an oncogene. This group demonstrated that the paradox was due to the occurrence of two forms of the *WT1* gene—the canonical *WTI* gene translated from an AUG start site (*augWT1*), acting as a tumor suppressor, and another form of the WT1 gene, translated from a CUG site (*cugWT1*), which has an extended N-terminal and appears to act as an oncogene.

Dr. John DiGiovanni discussed his work on novel approaches for targeting amino acid metabolism for the prevention and treatment of prostate cancer. Using a combination of high throughput screening techniques and mouse models, his group discovered that targeting tumor metabolic activity by inhibiting amino acid enzymes like cysteinase and a methioninase, could attenuate tumor growth in allograft and xenograft tumor models and increase survival in a mouse model of chronic lymphocytic leukemia.

Dr. Stephen Hecht discussed work supported by a program project grant on the elucidation of biomarkers that could help identify smokers at higher risk for lung cancer. Utilizing data from previous large scale studies, collaborators in this grant, including Sharon E. Murphy, Lani Park, Loic Le Marchand, Daniel Stram, and Yesha Patel, identified variants of the gene *CYP2A6* (a major nicotine metabolizing enzyme) as biomarkers for lung cancer risk. Low metabolizing alleles of *CYP2A6* were associated with a lower lung cancer risk, because of a decreased need for unchanged nicotine.

### Advances in targeted cancer therapies

More efficient targeting of carcinogenesis could lead to improved therapies and outcomes, and several conference speakers provided insights into both the development of better therapeutic targets and novel targeting approaches.

Dr. Luke Hoeppner presented his studies targeting dopamine and cyclic AMP-regulated phosphoprotein (DARPP-32) and its splice variant t-DARPP in lung cancer. Overexpression of DARPP-32 and t-DARPP promoted lung tumor growth in human xenograft orthotopic mouse models by stimulating lung cancer cell survival and migration. Dr. Hoeppner’s group also found that elevated t-DARPP expression correlated with worsening tumor grade and decreased non-small cell lung cancer patient survival. Thus, DARPP-32 signaling could be a new potential molecular target for lung cancer therapy.

Dr. Ming You presented work on using the natural compound honokiol (HNK), a magnolia tree extract, for targeting lung cancer. Mitochondria were recently discovered as a key target of HNK, so Dr. Ming’s group synthesized a mitochondrial-targeted HNK (Mito-HNK). Using mouse models, they found that Mito-HNK was substantially more potent than HNK in inhibiting the proliferation and invasion of lung cancer cells as well as attenuating lung cancer progression and metastasis. Also, Mito-HNK has a favorable toxicity profile making it an attractive compound for treating lung cancer.

Dr. Sergio Gradilone discussed his work on primary cilia and their potential as therapeutic targets for carcinogenesis. The role of cilia in tumorigenesis is unclear, but loss of primary cilia occurs in many cancers. Reduction of cilia is associated with overexpression of the HDAC6 protein, which is also associated with increased cancer cell proliferation. Dr. Gradilone’s group showed that inhibiting HDAC6 by a drug or knock-down restored cilia and reduced proliferation. This innovative approach could help develop novel and effective therapies.

Dr. Richard Eckert presented work on epidermal cancer stem (ECS) cells. ECS cells drive the formation of rapidly-growing, highly invasive and vascularized tumors. Dr. Eckert’s group identified a new signaling cascade with the neurophilin 1 (NRP1) protein, which contributes to tumor vascularization, and could therefore be a novel therapeutic target. NRP-1 knockdown reduced the size and vascularization of tumors in tumor xenograft experiments.

### Science publishing in the 21st century

The merger between Springer and Nature has created the largest worldwide portfolio of open access journals. Andrea Macaluso from Springer Nature discussed the role of open access journals in the science publishing landscape. The new open access journal *npj Precision Oncology*, a collaboration between Springer Nature and The Hormel Institute, University of Minnesota, was highlighted.

## Conclusions

The conference provided an opportunity for diverse cancer researchers to come together and discuss many aspects of cutting-edge science. The hope is that the insightful presentations and energetic scientific discussions among the researchers, will spur more collaborations and improve our understanding of the diverse aspects of cancer biology.

## Permissions

Presenters provided their permission to publish details about their titles and summaries in the table and text.

## Ethical approval

All animal and human research was approved by and conducted in accordance to relevant institutional ethics board standards.

## Informed consent

All human patients had informed consent
